# Immune and Inflammatory Cells of the Tumor Microenvironment Represent Novel Therapeutic Targets in Classical Hodgkin Lymphoma

**DOI:** 10.3390/ijms20215503

**Published:** 2019-11-05

**Authors:** Eleonora Calabretta, Francesco d’Amore, Carmelo Carlo-Stella

**Affiliations:** 1 Department of Oncology and Hematology, Humanitas Cancer Center, Humanitas Clinical and Research Center, Rozzano, 20089 Milano, Italy; 2 Department of Haematology, Aarhus University Hospital, 8200 Aarhus, Denmark; francesco.damore@clin.au.dk; 3 Department of Biomedical Sciences, Humanitas University, Rozzano, 20089 Milano, Italy

**Keywords:** classical hodgkin lymphoma, microenvironment, immune evasion

## Abstract

Classical Hodgkin Lymphoma (cHL) is a B-cell malignancy that, typically, responds well to standard therapies. However, patients who relapse after standard regimens or are refractory to induction therapy have a dismal outcome. The implementation of novel therapies such as the anti-CD30 monoclonal antibody Brentuximab Vedotin and immune checkpoint inhibitors has provided curative options for many of these patients. Nonetheless, responses are rarely durable, emphasizing the need for new agents. cHL is characterized by a unique microenvironment in which cellular and humoral components interact to promote tumor survival and dissemination. Knowledge of the complex composition of cHL microenvironment is constantly evolving; in particular, there is growing interest in certain cell subsets such as tumor-associated macrophages, myeloid-derived suppressor cells and neutrophils, all of which have a relevant role in the pathogenesis of the disease. The unique biology of the cHL microenvironment has provided opportunities to develop new drugs, many of which are currently being tested in preclinical and clinical settings. In this review, we will summarize novel insights in the crosstalk between tumor cells and non-malignant inflammatory cells. In addition, we will discuss the relevance of tumor-microenvironment interactions as potential therapeutic targets.

## 1. Introduction

With an incidence of 2.4 cases per 100,000 persons in Europe [1], Hodgkin lymphoma (HL) represents approximately 10% of newly diagnosed lymphomas [2], and is one of the most common malignancies in young adults. Based on morphological and immunophenotypical features, HL can be divided in two major subgroups: classical Hodgkin lymphoma (cHL) which accounts for approximately 95% of cases, and nodular lymphocyte- predominant HL (NLPHL) which accounts for the remaining cases. The latter is, however, considered a separate disease entity, as malignant cells retain B cell surface antigens and germinal center-specific markers [3,4].

By contrast, cHL is characterized by a unique histological appearance, consisting of few neoplastic cells–the so-called Hodgkin and Reed-Sternberg (HRS) cells—embedded in a rich inflammatory infiltrate. Reed-Sternberg cells, which account for only 1–2% of the tumor, are large bi- or multi-nucleated cells, characterized by the expression, among others, of the CD15 and CD30 antigens. Although a common origin from germinal center B cells has been demonstrated [5], HRS cells are devoid of the typical B lymphocyte markers (CD20, BCL6, B cell receptor) and do not express immunoglobulins. The inflammatory infiltrate is extremely heterogenous and, based on its composition, cHL can be subdivided into four histological subtypes: nodular sclerosis, mixed cellularity, and the less common lymphocyte-rich and lymphocyte-depleted subtypes [6]. 

In approximately 40% of cHL patients the HRS are infected by the Epstein-Barr virus (EBV), which likely contributes to the pathogenesis of the disease; indeed, latently expressed viral proteins mimic B cell growth signals, therefore rescuing cells otherwise destined to undergo apoptosis [7]. EBV-positive cHL is generally associated with a poorer prognosis, and plasma EBV-DNA is considered an independent predictor of treatment failure [8].

In the last decades, combination of chemotherapy and radiotherapy has achieved excellent long-term outcomes, with up to 80% of patients affected by cHL cured by first-line therapy [9]. However, 20–30% of advanced stage patients are refractory to standard regimens or relapse shortly thereafter. 

High-dose salvage therapies, which aim at complete disease remission and subsequent consolidation with autologous stem cell transplantation (ASCT), have variable toxicity profiles and high response rates with an average 50% to 60% of patients receiving second-line chemotherapy and ASCT being cured [10,11,12,13]. The recent availability of brentuximab vedotin [14,15] and programmed cell death-1 (PD-1) checkpoint inhibitors [16] has significantly expanded the therapeutic armamentarium, thereby providing curative options for a proportion of relapsed/refractory cHL. Nevertheless, primary refractory and early relapsed patients as well as those who acquire chemorefractoriness still represent an unmet medical need, thus emphasizing the need to develop new therapeutic approaches. 

In this context, the complex interactions between HRS cells and the tumor microenvironment are being extensively investigated, with the goal of understanding the role of the abundant inflammatory cells in this peculiar disease [17]. Therapies aiming at augmenting the immune response have demonstrated extraordinary results in patients who failed all standard therapies [16,18], suggesting that dysregulations of the immune system may be more relevant than previously thought in the pathogenesis of the disease. 

This review aims at summarizing the existing knowledge on the crosstalk between neoplastic cells and microenvironment and highlighting the novel insights in the biology of cHL. In addition, we will focus on new therapeutic approaches exploiting the modulation of immune pathways.

## 2. The Tumor Microenvironment: Cellular Composition

As mentioned above, the bulk of the tumor in cHL is composed of many different types of non-malignant inflammatory and mesenchymal cells, including T and B cells, macrophages, neutrophils, eosinophils, mast cells, plasma cells and fibroblasts. The exact role of this cellular infiltrate is yet to be defined, however there is evidence that HRS cells actively inhibit the cytotoxic activity of immune cells and modulate their signaling pathways to promote a pro-tumoral microenvironment [19]. In turn, reactive cells produce cytokines and chemokines that allow HRS cells to survive, proliferate and evade antitumor immune mechanism. Additionally, genetic abnormalities cooperate with microenvironment-dependent signaling in the activation of the pro-tumoral pathways NF-kB and JAK-STAT. In this complex network of interactions, the balance between microenvironmental growth signals and immune escape strategies is what ultimately determines tumor growth [20].

### 2.1. T cells

In cHL, the dominant cell type are T lymphocytes, specifically CD4-positive (CD4+) helper T cells and regulatory T cells (Tregs), which are attracted by HRS cells via secretion of the CCL5, CCL17, and CCL22 chemokines [21]. Crosstalk between HRS cells and CD4-positive T cells is mediated primarily by the CD40-CD40 ligand interaction, leading to the activation of the NF-kB pathway, which has a crucial role in promoting tumor growth and disease progression [22]. 

Effective anti-tumor activity is dependent upon CD4+ T cells, which orchestrate many immunological pathways that ultimately lead to effector activation. Two predominant T helper cell subtypes exist, T helper 1 (Th1) and T helper 2 (Th2). Th1 cells are responsible for activating antigen-presenting cells and cytotoxic T cells; in addition, such cells display a certain degree of direct cytotoxic activity. On the contrary, Th2 cells favour tumour growth, both by promoting angiogenesis and by inhibiting cell-mediated immunity and subsequent tumour cell killing.

Initial studies reported a prevalence of Th2 lymphocytes in the tumor infiltrate of cHL, which would be consistent with a hypofunctioning host immune response against the tumor [23]. However, more recent studies have demonstrated a predominance of Th1 pro-inflammatory cells [24], which appear to be poorly effective in clearing malignant cells. The potential mechanisms leading to reduced T cell function will be discussed in detail in the next section. 

cHL is exceptionally rich in Tregs compared to other hematological and non-hematological malignancies [25]. These cells actively inhibit cytotoxic activity via numerous mechanisms, some of which are still unknown or poorly understood. Recognized mechanisms include IL-10 secretion, CTLA-4 upregulation, and overexpression of LAG-3, which in turn down-regulates CD3 T-cell receptor mediated signaling [26,27]. Among the various subtypes, T regulatory 1 cells are the most prevalent, and act specifically to inhibit Th1 helper effector cells [25,28]. Though their contribution to the reduced anti-tumor response is likely pivotal, large quantities of Tregs in cHL tissues are not associated with an adverse prognosis [29,30].

The few neoplastic cells rely significantly on the presence of T cells in order to survive; indeed, in vivo studies in immunodeficient mice demonstrated the death of RS cells, when not supported by helper T cells [31]. This vital relationship, which is physically manifested by formation of HRS-T cell aggregates, the so-called ‘rosettes’ [32], is regarded as one of the hallmarks of cHL.

### 2.2. Tumor-associated macrophages

In a wide variety of malignancies, macrophages drive tumor related inflammation, thus contributing to tumor progression, and are strong promoters of angiogenesis and lymphogenesis [33]. Tumor-associated macrophages (TAMs) are abundant in the microenvironment of cHL. Interaction with HRS cells, as well as exposure to soluble factors such as IL-4, IL-13 and M-CSF [34], leads to a polarization of macrophages towards the M2 phenotype, characterized by active secretion of anti-inflammatory cytokines such as IL-10, TGF-β and PGE2 [35]. M2 macrophages suppress cytotoxic T cell activity and attract Tregs, therefore facilitating tumor growth and immune escape. Interestingly, TAMs are more frequent in EBV+ cHL than in EBV-, though the exact mechanism underlying this difference remains obscure. TAMs in EBV+ cHL also seem to be strongly associated with the expression of CD163 suggesting the presence of a predominantly M2-like phenotype [36].

High numbers of TAMs, as identified by the marker CD68, in lymph node biopsies have been associated with decreased survival and poor treatment outcome, particularly after secondary therapies such as ASCT [37,38,39]. However, subsequent studies have yielded conflicting results [36,40,41]. A possible explanation may be that expression of CD68 is not restricted to TAMs. Therefore, Klein and colleagues have proposed to monitor the expression of the CD163 antigen, which appears to be a more reliable prognostic marker [42]. 

Nonetheless, macrophages are undeniable contributors to the interplay between HRS cells and microenvironment. The predominant M2 phenotype has a cardinal role in the pathogenesis of the disease and represents a potential therapeutic target. Likewise, modulating the macrophage phenotype towards a pro-inflammatory M1-like may aid in the formation of a more effective immune surveillance system in cHL [43].

### 2.3. Myeloid-derived Suppressor Cells and Neutrophils

An exciting new field in cancer research is represented by a class of myeloid cells which exhibit an immunosuppressive behavior and are referred to as myeloid-derived suppressor cells (MDSCs). MDSCs are a heterogeneous population of activated immature myeloid cells in different stages of differentiation that are characterized by the expression of markers commonly associated with granulocytes [44,45]. These cells undergo expansion and accumulate in many cancer types, where they promote tolerance and immune escape, as well as tumor dissemination [46]. Their exact immunophenotypical signature is still controversial, as different subsets are more prevalent in different tumor types [47,48,49]. Populations of MDSCs have been identified also in the peripheral blood of Hodgkin and Non-Hodgkin lymphoma (NHL) patients; they appear to correlate with disease aggressiveness and have a strong prognostic significance [50,51]. Accurate identification and characterization of MDSCs directly in lymph-node biopsies may aid in identifying the most relevant subpopulations and potential therapeutic targets.

Recently, researchers have demonstrated that also mature granulocytic cells, i.e., neutrophils, exert an immunosuppressive function in cancer patients [52]. These cells, which may be found in the tumor microenvironment, the so-called tumor-associated neutrophils (TANs), interact with tumor cells to promote angiogenesis, cancer cell invasion and metastases [53,54,55,56]. Indeed, the peripheral blood neutrophil-lymphocyte ratio has been implicated as an independent prognostic factor in many malignancies, suggesting that neutrophil-induced inflammation is a key component in cancer progression. Its prognostic significance was also confirmed in cHL [57,58]. However, the role of neutrophils may not be so clear-cut: indeed, Ponzetta et al. have recently demonstrated that, in certain tumors, TANs promote an IFN-γ-mediated anti-tumoral response mediated by unconventional CD4-CD8- T cells [59]. This novel insight further highlights the diversity and complexity of tumoral microenvironment and may warrant further investigations also in lymphomas. 

It has been suggested that certain subtypes of MDSCs derive from a pathological activation of neutrophils, driven by microenvironmental signals. A recent study from Veglia et al. has identified in alterations of the lipid metabolism as possible contributing factors in their pathological activation. Indeed, selective inhibition of fatty acid transport protein 2 (FATP2) resulted in decreased MDSC activity and delayed tumor progression in preclinical murine models of lung cancer [60]. Additionally, an increased MDSC inhibition was noted in the context of checkpoint inhibition, suggesting an interaction with other immune cells of the microenvironment.

Collectively the role of neutrophils and MDSCs has become increasingly relevant in the past decade in cancer biology. Targeting of MDSCs and reprogramming the phenotype of TANs represent new therapeutic opportunities for cHL refractory patients, though human studies have yet to be elaborated.

### 2.4. Eosinophils and Mast Cells

cHL tissues are rich in eosinophils and mast cells. Many studies have found a correlation between the abundance of such cells and an inferior prognosis [61,62,63]. Interestingly, no impact has been observed in the paediatric population [64]. The number of eosinophils and mast cells is influenced by some host factors, such as history of asthma, polymorphisms of eosinophilic cationic protein and EBV- status [65]. However, the biological mechanisms underlying the adverse outcome are largely unknown, and may thus provide therapeutic opportunities. 

### 2.5. NK cells

Despite the dominance of immuno-suppressive cellular populations (Tregs, Th2 helper cells, M2 macrophages and MDSCs), cytotoxic cells, such as CD8-positive T cells, Th1 helper cells and natural killer (NK) cells, are still present in the tumor microenvironment. NK cells are attracted to sites of disease by various chemokines and cytokines, such as CXCL9, CXCL10 and interferon (IFN)-γ. However, they are quantitatively and functionally deficient [66]. Among the factors responsible for impaired killing capacity, a major role is represented by the absence of IL-2, the main activating cytokine of NK cells, due to production of soluble IL-2 receptor (CD25) by HRS cells, which functions as decoy competing for cytokine engagement [67,68,69]. 

In healthy individuals, most circulating NK cells are CD56^dim^ CD16^postitive^, characterized by highly effective direct and antibody-mediated cytotoxic activity. Conversely, in the peripheral blood of cHL patients, most NK cells are CD56^bright^–CD16^negative^ [70], and exhibit a cytokine-mediated anticancer activity. However, HRS cells seem to be resistant to this type of killing mechanism, the reasons of which are still being investigated. Thus, promoting NK cell activation and rendering HRS cells susceptible to NK-mediated killing represent important areas of investigation for the development of novel and more effective therapeutic strategies.

### 2.6. B cells

The tumor microenvironment is also rich in non-malignant reactive B cells, whose role is still poorly defined. High numbers of CD20+ B cells within the microenvironment have been associated with an improved survival [37]. A possible explanation for this finding may rest on competition with malignant cells for growth signals. In this context, attempts at targeting B cells with Rituximab may appear counter-intuitive. Nonetheless, initial studies reported improved progression-free survival (PFS) in cohorts of patients treated with the anti-CD20 monoclonal antibody, alone or in combination with first-line therapies, and regardless of CD20 expression [71,72]. The survival benefit may be explained by targeting HRS precursor cells, which retain B cell markers, or by suppressing survival signals from reactive non-neoplastic B cells. Such results, however, were recently refuted: indeed, the use of Rituximab-ABVD in high-risk patients [73] and Rituximab-BEACOPP in interim PET-positive patients [74] failed to produce an improved survival. Based on these results, Rituximab is no longer considered as an adjunctive treatment option in cHL; however, the role of the rich B cell infiltrate still needs to be clarified, and may aid in providing additional therapeutic targets.

### 2.7. Fibrobalsts and extracellular matrix

Finally, we must not forget that all these cell types reside within an extracellular matrix (ECM) composed of collagen and reticular fibers produced by fibroblast-like cells [75]. Formation of fibrotic tissue relies on production of TGF-β, b-FGF and IL-13 by reactive cells and HRS cells. In turn, fibroblasts secrete molecules which may be involved in cell survival and proliferation, such as IL-6 and IL-7 [76]. In this scenario, extracellular vesicles, which stimulate fibroblasts to produce pro-inflammatory, pro-tumoral and angiogenetic molecules, have been recently found to mediate crosstalk between different cell types, thus playing a fundamental role in shaping the tumor microenvironment [77]. Further studies are warranted to fully appreciate the biological potential of fibroblast-like cells and the ECM.

## 3. Evading Immune-surveillance: How HRS cells Use the Microenvironment to Their Advantage

During tumorigenesis, HRS cells develop various strategies to attract non-malignant inflammatory cells to their surroundings. Intense crosstalk allows the formation of a unique niche, in which anti-tumor activity is suppressed and cellular composition and functional activity is shaped based on the necessities of malignant cells. Many strategies of immune-evasion have been identified including: (i) programmed-death 1 (PD-1) overexpression and signaling; (ii) activation of the JAK/STAT pathway; (iii) inactivating mutations of human leukocyte antigen (HLA); (iv) overexpression of tolerogenic surface molecules; and (v) creation of an immunosuppressive cytokine network.

PD-1 is an inhibitory receptor which is expressed by activated T cells. Its ligands, PDL-1 and PDL-2, are present on the surface of antigen-presenting cells. Interaction between these two components leads to activation of various intracellular pathways, which ultimately result in inhibition of T cell receptor signaling and downregulation of cytokine production [78]. In a physiological context, this system represents a necessary brake to the activation of the immune system during inflammation. However, in cHL, as in many other tumors, PDL-1 is overexpressed both by malignant and surrounding reactive cells, greatly depressing cytotoxic anti-tumor activity of the adaptive immune system, in a process which is termed “T-cell exhaustion”. A genetic basis for this phenomenon was identified by Green and colleagues: they were able to detect an amplification of 9p24.1, in correspondence of the PDL-1 gene and, to a lesser extent, PDL-2 gene, in HRS cells from nodal biopsy samples [79]. Additional analyses have revealed a gain of a copy of the JAK2 gene just upstream of 9p24.1, in the same amplicon; enhancement of the JAK-STAT pathway has therefore been proposed as a mechanism for inducing PD1 ligand expression. Indeed, 9p24.1 amplification negatively impacts PFS in patients with newly diagnosed cHL [80]. Lastly, the same research group was able to demonstrate that EBV infection also induces PDL-1 upregulation [81], therefore revealing additional biological mechanisms of tumor survival. Accordingly, it is reasonable to speculate that EBV+ cHL patients may especially benefit from checkpoint blockade. Recently, Anastasiadou et al., have demonstrated that the viral protein EBNA2 induces PDL-1 in EBV+ diffuse large B cell lymphoma by downregulating miR-34a [82]; although EBNA2 is not expressed in EBV-associated cHL, it may have been expressed at some stage of tumor development and subsequently switched off.

As proof of the essential bond between HRS cells and tumor microenvironment, recent studies have revealed a synergistic action of non-malignant cells in promoting PD-1 signaling. Indeed, TAMs expressing PDL-1 as a consequence of high IFN-γ concentration were found in the proximity of PDL-1+ HRS cells [83]. Vari et al. have demonstrated that these PDL-1+ macrophages primarily target and suppress NK cells, which express high levels of PD-1 [70]. This finding has clarified a possible mechanism of inefficient NK cell cytotoxic activity in cHL, along with providing an additional biological basis for the impressive efficacy of therapies with PD-1 blockers, which can revert the “NK cell exhaustion” mediated by the interaction of PD-1+NK cells and PD-L1+ monocytes/macrophages. Analysis of PD-1 expression in tumor infiltrating T cells is heterogeneous among different studies, due to a variety of factors including differences in the binding efficacy of different PD-1 monoclonal antibodies [84]. This finding makes it difficult to speculate on the mechanisms underlying the exceptionally high efficacy of PD-1 inhibitors. A possible explanation for this success may be found in the presence of other microenvironmental components who express PD-1, such as NK cells [70], who likely contribute to the anti-tumoral response.

An additional mechanism leading to immune-evasion is the inability of T cells to recognize malignant cells. This is mainly due to lack expression of major histocompatibility complex (MHC) I [85] on HRS cells, which is necessary for recognition by cytotoxic CD8+ T lymphocytes. Whole-wide genome analyses have shown that the main molecular mechanism responsible for the downregulation of MHC I is an inactivating mutation of the β-2-microglobulin chain, especially occurring in the nodular-sclerosis variant [86]. Surprisingly, EBV-associated cHL retains or even overexpresses MHC I [85], suggesting that HLA downregulation represents only one of many possible mechanisms of immune escape. Lack of HLA class I may expose HRS cells to NK-mediated killing; malignant cells overcome this liability by depressing cytotoxic activity via secretion of inhibitory molecules, as previously described, and by upregulating HLA-E and HLA-G. These specific types of MHC I molecules interact with inhibitory receptors on NK cells, thus “naturally” suppressing anti-tumor activity [87]. Interestingly, MHC I loss was almost always detected in the presence of PDL-1 upregulation: CD8+ T cells cannot, therefore, exert their cytotoxic activity after PD-1 blockade, raising questions on which cells may replace them. The role of CD4+ cells and the innate immune system is currently under investigation in this setting. Approximately 15% of cHL patients concurrently exhibit downregulation of the HLA class II due to translocations involving the MHC class II transactivator (CIITA) gene locus [88]. Lack of HLA class II expression by HRS cells is considered an independent adverse prognostic factor in cHL [89].

Another mechanism contributing to immune-evasion is the upregulation of surface molecules, such as Fas ligand and galectin-1, which modulate T-cell immunity. Virtually all HRS cells express the death receptor Fas (CD95) but resist Fas ligand-mediated apoptosis by expressing the FLICE inhibitory protein (cFLIP) [90]. Interestingly, c-FLIP expression shields from apoptosis, but does not inhibit CD95-mediated signaling on HRS, which results in activation of NF-κB, the major proliferative stimulus of HL [91]. Additionally, approximately 30% of HRS cells express FAS ligand (CD95L) [92] that may induce apoptosis of activated, FAS expressing, CD8+ T cells and NK cells [93].

Galectin-1 (Gal-1) is a soluble beta-galactoside binding lectin which is known to promote apoptosis of active T cells [94] and potentiate Treg activity [95]. A study by Gandhi et al. detected Gal-1 overexpression in approximately 60% of cHL; Gal-1 positive patients had a reduced CD8+ T cell infiltrate and impaired antigen-specific T cell responses [96]. Indeed, Gal-1 levels are now a recognized prognostic factor, and correlate with tumor burden, potentially aiding in identifying high-risk subpopulations [97,98].

Lastly, HRS cells are able to shield themselves from the attack of cytotoxic T cells by secreting immune suppressive cytokines, such as IL-10 and TGF-β, and soluble factors, such as prostaglandin E2 (PGE2), which suppress the activity of effector cells. IL-10 is a potent immune suppressive cytokine, which is actively produced by HRS cells and surrounding reactive cells. Its levels are greatly elevated in up to a half of cHL patients and are correlated to disease aggressiveness and poor response to therapy [99]. Likewise, TGF-β production contributes to depress T-cell proliferation, cytokine release, and cytolytic activity. Finally, PGE2 has also been implicated as a contributor to immunosuppression and pathogenesis of cHL; PGE2 interferes with T cell receptor signaling, therefore weakening the cytotoxic response [100]. Recently, Wein et al. have also identified an increased adenosine signaling, which inhibits pro-inflammatory activity of Th1 cells and NK cells and can activate immune-suppressive Tregs and MDSCs, adding to the complexity of molecular signaling in the cHL microenvironment [101].

In conclusion, ongoing research has identified several cellular and molecular mechanisms that collectively allow malignant cells to resist attack from host innate and adaptive defenses. Our understanding of the composition of cHL microenvironment has progressively become more refined; however, the role of many components and their interaction with malignant cells remains unexplored. Clearly, Hodgkin and Reed Steinberg cells have developed an extraordinary ability to orchestrate the formation and persistence of an environment which can promote tumor growth while preventing and blocking many anti-tumoral responses.

## 4. From Bench to Bedside: Therapeutical Potential of Tumor-microenvironment Interaction

**Figure 1:**
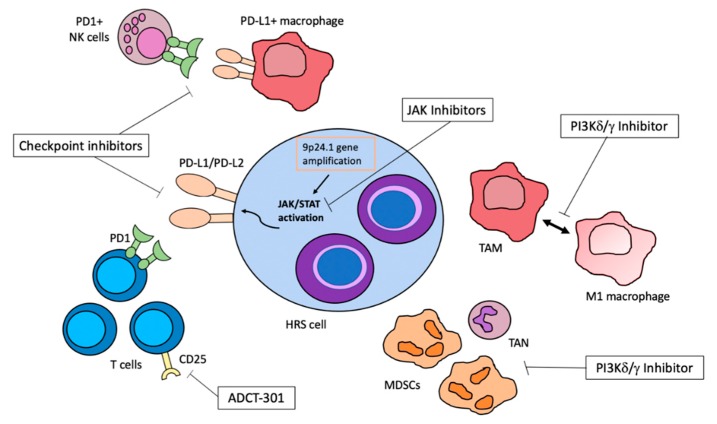
A schematic representation of novel therapeutic targets in classical Hodgkin lymphoma (cHL), exploiting tumor–microenvironment interactions. The black arrow represents a bimodal shift in macrophage polarization.

The importance of fully understanding the multiple signaling pathways and mechanisms of cellular interaction is to identify potential therapeutic targets for patients with refractory or relapsed cHL, as well as to reduce therapy-related toxicity of first-line regimens. Chemo-refractory patients have a poor prognosis [102,103]. Thus, exploring other therapeutic options is a current challenge to improve survival in this subset of patients and therapies targeting inflammatory cells or microenvironmental signals have shown encouraging results. Here, we review the most promising agents, which are summarized in Figure 1 and Table 1.

### 4.1. Checkpoint Inhibitors

The recent discovery of widespread expression of PDL-1 and PDL-2 on HRS cells and reactive cells of the microenvironment, and concomitant PD-1 upregulation on the surface of intratumoral T cells, has paved the way for checkpoint blockade and many other immunotherapy-based trials in relapsed/refractory cHL. As chromosome 9p24.1 amplification involves both PDL-1 and PDL-2, it seemed initially more reasonable to target the shared receptor, PD-1. A pilot study carried out by Ansell et al. on 23 heavily pretreated patients reported an overall response rate of 87% after administration of Nivolumab, a fully human monoclonal IgG4 antibody directed against PD-1 [16]. A subsequent multicenter phase II study (CheckMate 205) demonstrated a response rate of 66% in patients who had relapsed after ASCT and BV [18]. Notably, Nivolumab was well tolerated: most patients experienced only low-grade toxicity and reported an improvement in quality of life. An extended follow-up of the patients included in this trial demonstrated durable responses at a median follow-up of 18 months and increased OS and PFS also in patients with stable disease (SD) [104]. Another humanized IgG4 monoclonal antibody targeting PD-1, Pembrolizumab, has shown response rates comparable to Nivolumab in the same patients’ population [105,111]. These studies led to the approval of such antibodies for the treatment of patients with relapsed and refractory HL, who otherwise have a dismal prognosis. However, long-term efficacy is still unknown, underlying the importance for these patients to receive allogeneic stem cell transplantation (allo-SCT) when feasible, to consolidate the response. In this setting, a multicenter study has demonstrated a considerable efficacy of allo-SCT, with low relapse rates and manageable transplant-related toxicity [112]. Additionally, ongoing efforts are directed to understand potential mechanisms of acquired resistance to checkpoint inhibitors. Chen et al., have identified upregulation of CD38 driven by infiltrating T cells, activated by PD-1/PDL-1 inhibition [113]. CD38 acts by stimulating adenosine signaling, which, in turn, exerts an immunosuppressive action on CD8+ T cells, thus providing escape from the activated adaptive immune cells. Indeed, in tumor-bearing mice, combination of anti-CD38 antibody and anti-PDL-1 antibody suppressed tumor growth and dissemination more than either antibody alone. The use of such combination in the clinical setting may aid in reducing resistance to checkpoint blockade.

Currently, ongoing trials are exploring the possibility of including PD-1 inhibitors in earlier stages of cHL treatment, in combination with standard chemotherapy or BV. Encouraging results have already been observed in advanced stage cHL treated with Nivolumab-AVD within the CheckMate 205 trial [114]. Additionally, PD-1 inhibitors are being successfully evaluated as initial salvage therapies in relapsed/refractory HL [115] and as consolidation after ASCT [116]. Upcoming results are greatly awaited and may reshape current standard regimens for cHL.

Despite concerns of leaving the PD-1/PDL-2 interaction intact, antibodies targeting PDL-1 also have been developed and clinically tested. Avelumab is a fully human IgG1 monoclonal antibody that selectively binds to PD-L1; Chen et al. investigated the efficacy of this antibody in a cohort of patients affected by relapsed/refractory cHL. Results were comparable to PD-1 blockade, with an overall response rate of 42%, suggesting that PDL-1 is the main inducer of T cell exhaustion [106].

Cytotoxic T-lymphocyte antigen 4 (CTLA-4) is exclusively expressed on T cells, and interaction with its ligand provides an inhibitory signal in the early stages of T-cell activation [117,118]. Its expression is augmented by T cell receptor activation and certain inflammatory cytokines, such as IFN-γ, providing a ‘natural’ brake to the activation of the adaptive immune system. Ipilimumab, a fully human IgG1 monoclonal antibody targeting CTLA-4, was the first checkpoint inhibitor to be approved for clinical use after remarkable results in advanced-stage melanoma [119]. This monoclonal antibody was tested also in patients affected by advanced hematological malignancies who had relapsed after allo-SCT: of 14 patients affected by HL, 3 achieved an objective response [120].

Collectively, such dramatic results have provided hope to cure many young patients who previously failed all available treatment options. The future direction is to incorporate these novel agents into first-line regimens in order to reduce the rates of relapse and refractoriness.

### 4.2. PI3K Inhibitors: Targeting TAMs and MDSCs

The molecular mechanisms underlying the pathogenesis of cHL are largely unknown. A major contribution to malignant transformation and proliferation of HRS cells consists in the activation of the NF-kB and JAK-STAT signaling pathways, as previously stated. Additionally, recent studies have focused on the contribution of phosphatidyl-inositide 3 kinase (PI3-kinase), and have demonstrated that Akt, a substrate of PI3-kinase, is constitutively activated in HL-derived cell lines [121].

The 4 class I PI3K isozymes (PI3Kα, PI3Kβ, PI3Kγ, and PI3Kδ) regulate a variety of cellular functions, and the δ isoform is specifically involved in B-cell signaling and regulates B-cell response to chemokines and cytokines [122,123]. In vitro exposure of HRS cells to a specific inhibitor of PI3Kδ resulted in increased levels of apoptosis [124]. Based on these findings, Idelalisib, a selective inhibitor of PI3Kδ, was administered to patients affected by relapsed/refractory cHL in a phase II study [107]: compared to other B-cell malignancies, the activity of Idelalisib in this patient population was modest, with only a 20% response rate.

Conversely, the PI3Kγ isoform is expressed especially on myeloid cells of the tumor microenvironment and is involved in secretion of anti-inflammatory cytokines that promote tumor escape [125]. De Henau et al. recently demonstrated in solid tumor mouse models that pharmacological PI3Kγ inhibition is capable of switching macrophages from an M2 phenotype to a more cytotoxic M1 phenotype, thus resulting in tumor regression [126]. Additional results also showed that modulation of macrophage activity may increase the sensitivity to checkpoint blockade. Moreover, based on recent data showing that EBV-derived LMP2A utilizes Syk and PI3K to activate NF-κB in B-cell malignancies, PI3Kγ inhibition may have additional relevance in EBV+ cHL [127]. As previously stated, cHL microenvironment is rich in TAMs. Therefore, such agents may represent an effective therapeutic option. Indeed, preclinical studies testing RP6530, a dual PI3Kδ/γ Inhibitor, on HL cell lines and mouse models revealed a reprogramming of macrophages to the M1 phenotype, resulting in decreased tumor growth as well as inhibition of angiogenesis [43]. Additionally, RP6530 administration resulted in reduction of tumor-infiltrating MDSCs and, accordingly, correlated with clinical outcome. Appropriately designed clinical trials using dual PI3K inhibitors in relapsed/refractory cHL patients are warranted to confirm the therapeutic potential of targeting and modulating the inflammatory background.

### 4.3. JAK Inhibitors

Constitutive activation of the JAK/STAT signaling pathway is crucial for the development of cHL. Persistent signal transduction is promoted both by genetic and chromosomal alterations [128,129,130], as well as signals from the tumor microenvironment [19]. Additionally, as previously stated, chromosome 9p24.1 amplification involving the JAK2 region increases the expression of PD-1 ligands, thus favoring immune escape. In this scenario, JAK 1/2 inhibition may represent an innovative alternative to suppress tumor growth. Ruxolitinib, an oral inhibitor of JAK1/2, was tested in 33 patients with relapsed/refractory cHL: despite the promising rationale, monotherapy with this agent failed to produce satisfactory results, with an ORR of only 9.4% [108]. Nonetheless, it was well tolerated, potentially allowing for combination therapy. Indeed, encouraging results have come from combination of Ruxolitinib with BV in a mouse model of HL [131] and of the JAK1 inhibitor Itacitinib with a PI3Kδ inhibitor in the clinical setting. In the latter study, drug combination produced effective and durable responses in 67% of patients compared to only 29% with the PI3Kδ inhibitor monotherapy, while remaining largely tolerable [109]. The reasons for such successful synergistic action are unclear, and further studies are warranted to appreciate the potential of this drug combination, which may represent an effective alternative for patients who fail checkpoint blockade.

### 4.4. Anti-CD25 Antibodies

The inflammatory background in cHL is mostly constituted by infiltrating T lymphocytes. Most of these cells express CD25, which is also frequently expressed on HRS cells [132], thus making it a suitable target. Initially, a phase I trial investigated the activity of radio-immunotherapy using CHT-25, an anti-CD25 antibody, conjugated to iodine in patients with relapsed Hodgkin and T cell lymphomas [133]. CHT-25 was well tolerated and showed promising clinical activity with 6 of 9 patients achieving an objective response. A subsequent phase II trial involved 40 patients affected by relapsed/refractory cHL to ^90^Y-daclizumab (an anti-CD25 antibody): the ORR was approximately 50% [134]. This is impressive efficacy is most likely related to direct targeting of the rosetting T cells, rather than HRS cells. On the basis of these results, a trial investigating escalating doses of ^90^Y-daclizumab followed by ASCT has been developed. Lastly, Horwitz et al. recently disclosed interim results from a phase I study of ADCT-301 (Camidanlumab Tesirine), an antibody drug conjugate comprising a human monoclonal antibody against CD25, in relapsed/refractory HL and NHL patients. Among the HL cohort, 71.4% of patients achieved a response, despite being heavily pre-treated and often failing treatment with checkpoint inhibitors [110]. These results have generated great excitement, but final outcomes have yet to be published.

## 5. Conclusions

In recent years, significant advance has been made in understanding the biology of HL. The abundant cellular and humoral components of the inflammatory infiltrate play a pivotal role in tumor formation, survival and dissemination. Each component of the microenvironment has an established role; any numerical and/or functional alteration may result in loss of crucial interactions that drive tumorigenesis and in inability to evade attack by host immune response.

Disruption of this highly specialized niche has provided opportunities for the development of novel therapeutic agents. Checkpoint inhibitors have an established efficacy and are already approved for treatment of relapsed/refractory cHL. Unfortunately, a high number of patients is refractory even to anti-PD1 agents. Alternative strategies are needed to improve outcome in these highly refractory and aggressive subsets of cHL. In this setting, targeting TAMs and CD25+ T cells or combining various agents targeting different components of the microenvironment may increase the possibility of obtaining disease remission. Nonetheless, studies with longer follow-up are still lacking and potential long-term effects are unknown, though preliminary results have shown manageable toxicity.

Additionally, no reliable biomarkers to predict response to these novel therapies are available. Identification of suitable and easily accessible biomarkers may aid in patient selection and avoid unnecessary toxicity. Likewise, incorporation of such therapies in first-line regimens needs also to be considered for future therapeutic approaches. Most patients affected by cHL are effectively cured by standard chemotherapy; however, short- and long-term side effects are not uncommon. Future hope is to reshape current chemotherapy-based regimens to reduce unwanted toxicity and cure a greater percentage of patients with first-line therapies. Our increasingly refined knowledge of the complex interactions between malignant cells and the surrounding infiltrate will most certainly aid in achieving such objectives.

In summary, it can be safely concluded that in cHL the role of the microenvironment is unique among malignancies. Indeed, unlike other cancer types, few pathogenetically relevant genetic lesions targeting growth and survival pathways have been identified. NF-kB pathway activation, which has a major role in tumorigenesis, is only rarely activated by direct genetic lesions [135], but is more commonly activated by interaction with tumor-infiltrating T cells. However, many genetic lesions indirectly promote tumorigenesis by modulating microenvironmental survival signals. Examples include: inactivating mutations of β-2-microglobulin, 9p24.1 amplification resulting in PDL-1 overexpression and immune escape, translocations involving the MHC CIITA gene and epigenetic alterations resulting in suppression of the B cell phenotype. Aside from providing understanding of the biology of cHL, such genetic lesions may have a role in response/resistance to immunotherapy, which, despite excellent short-term effects, often fails to generate long-term control of the disease. In this scenario, liquid biopsy technique may allow to define the whole mutational landscape and detect mutations that determine disease behavior as well as response to therapy [136].

## Figures and Tables

**Table 1:** Efficacy of novel agents for the treatment of relapsed/refractory cHL.

Authors	Drug	Patients (*N*)	OS/PFS/ORR	References
Armand et al., J Clin Oncol 2018	Nivolumab	243	Median PFS 14.7 months Median OS not reached (18 months median FU)	[104]
Chen et al., Blood 2019 [abstract]	Pembrolizumab	210	Median PFS 16.5 months Median OS not reached (2 years median FU)	[105]
Chen et al. [abstract Lugano conference 2017]	Avelumab	31	ORR 54%	[106]
Gopal et al., Ann Oncol 2017	Idelalisib	25	Median PFS 2.3 months Median OS 19.8 months	[107]
Van den Neste et al., Haematologica 2018	Ruxolitinib	33	ORR 9.4%	[108]
Phillips et al., Blood 2018	Itacitinib + PI3Kδ inhibitor INCB040093	21	51% PFS at 12 months	[109]
Horwitz et al. [abstract ASH 2017]	Camidanlumab Tesirine	35 *	ORR 71.4%	[110]

FU = follow-up, OS = overall survival, PFS = progression-free survival, ORR = overall response rate, * including patients previously treated with checkpoint inhibitors.
